# Antibodies Are Major Drivers of Protection against Lethal Aerosol Infection with Highly Pathogenic *Burkholderia* spp.

**DOI:** 10.1128/mSphere.00674-18

**Published:** 2019-01-02

**Authors:** Robert J. Hogan, Eric R. Lafontaine

**Affiliations:** a Department of Infectious Diseases, College of Veterinary Medicine, University of Georgia, Athens, Georgia, USA; b Department of Veterinary Biosciences and Diagnostic Imaging, College of Veterinary Medicine, University of Georgia, Athens, Georgia, USA

**Keywords:** *Burkholderia*, glanders, humoral immunity, melioidosis, vaccines

## Abstract

Burkholderia pseudomallei and Burkholderia mallei are the causative agents of melioidosis and glanders, respectively. There is no vaccine to protect against these highly pathogenic bacteria, and there is concern regarding their emergence as global public health (B. pseudomallei) and biosecurity (B. mallei) threats. In this issue of *mSphere*, an article by Khakhum and colleagues (N. Khakhum, P. Bharaj, J. N. Myers, D. Tapia, et al., mSphere 4:e00570-18, 2019, https://doi.org/10.1128/mSphere.00570-18) describes a novel vaccination platform with excellent potential for cross-protection against both *Burkholderia* species. The report also highlights the importance of antibodies in immunity against these facultative intracellular organisms.

## Abstract (precis)

Burkholderia pseudomallei and Burkholderia mallei are the causative agents of melioidosis and glanders, respectively. There is no vaccine to protect against these highly pathogenic bacteria, and there is concern regarding their emergence as global public health (B. pseudomallei) and biosecurity (B. mallei) threats.

## COMMENTARY

Burkholderia pseudomallei and Burkholderia mallei are closely related bacteria causing fatal infections in humans and animals. B. pseudomallei is commonly found in wet soils of countries bordering the equator and causes the global emerging tropical disease melioidosis (1–3). B. mallei is a host-adapted clone of *B. pseudomallei* that does not persist in the environment outside its natural equine reservoir. The organism causes the extremely contagious and incapacitating zoonosis glanders, which is a reemerging biosecurity threat closely monitored by the World Organization for Animal Health (4–6). Comparative analyses indicate that *B. mallei* evolved from *B. pseudomallei* through genomic reduction, and the genes retained by *B. mallei* have an average identity of 99% with *B. pseudomallei* orthologs (7–10). The clinical and pathological manifestations of disease caused by the organisms are also strikingly similar. In humans, infection typically occurs through punctured skin or the respiratory route, and the most common manifestations are life-threatening pneumonia and bacteremia (1, 6, 11, 12). Pathogenicity is complex and involves the coordinated expression of many virulence factors supporting extracellular and intracellular replication as well as dissemination to target organs (lungs, spleen, liver, lymph nodes) where *B. pseudomallei* and *B. mallei* form hallmark chronic lesions (13–16). Melioidosis and glanders are difficult to diagnose and require prolonged therapy with low success rates due in large part to intrinsic resistance of the organisms to antibiotics (17, 18). No vaccine exists to protect humans or animals, and there is concern regarding adversarial use given that *B. mallei* has previously been utilized as a biological warfare agent (6). For these reasons, the U.S. Federal Select Agent Program classifies *B. pseudomallei* and *B. mallei* as Tier 1 organisms, and the availability of medical countermeasures is considered a critical unmet need. Fortunately, the genetic, biochemical, and virulence similarities between *B. pseudomallei* and *B. mallei*, and the resemblance of the diseases they cause, suggest the feasibility of developing countermeasures that protect against both organisms.

Protection against aerosol infection is of particular interest, as it is one of the most common inoculation routes in natural cases and the most likely portal of entry for *B. pseudomallei* and *B. mallei* in the event of adversarial use. The current benchmark animal model to evaluate countermeasures is the mouse, especially the BALB/c (highly sensitive) and C57BL/6 (sensitive) strains. The model produces hallmarks of melioidosis and glanders (low infectious and lethal doses, rapid bacterial replication in the lungs, dissemination to deep tissues, and formation of chronic lesions), and infected mice produce antibodies against antigens known to be targets of the human immune response, thus demonstrating immunological parallels (19–26). A number of experimental vaccines have been tested using the model, but none achieve complete protection and sterile immunity (27–29). Best-in-class vaccines afford increased survival against lethal challenge but do not prevent persistence of the organisms; mice develop lesions with high tissue burden and succumb to chronic infection despite possessing humoral and cellular immunity against *B. pseudomallei* and *B. mallei*. This failure to eliminate infection is a major obstacle in the field and emphasizes the need to expand the current pool of *Burkholderia* antigens for vaccine generation and to develop efficacious vaccination platforms.

In this issue of *mSphere*, a study by Khakhum and colleagues (30) demonstrates that immunization of C57BL/6 mice with a novel *B. pseudomallei* live attenuated strain (LAS) results in remarkable protection against lethal aerosol challenge with homologous wild-type bacteria. Khakhum et al. show that LAS vaccination elicits robust humoral and cellular immune responses, provides 100% survival for a period of up to 27 days after infection with highly pathogenic *B. pseudomallei* strain K96243, and results in outstanding rates of bacterial clearance in the lungs, liver, and spleen (71%). Importantly, they demonstrate through depletion experiments that protection is primarily dependent on humoral immunity. Their data indicate that 16 days postchallenge, mice vaccinated with LAS and subsequently depleted of CD4^+^ and CD8^+^ T cells show 60% and 100% survival, respectively.

**FIG 1:**
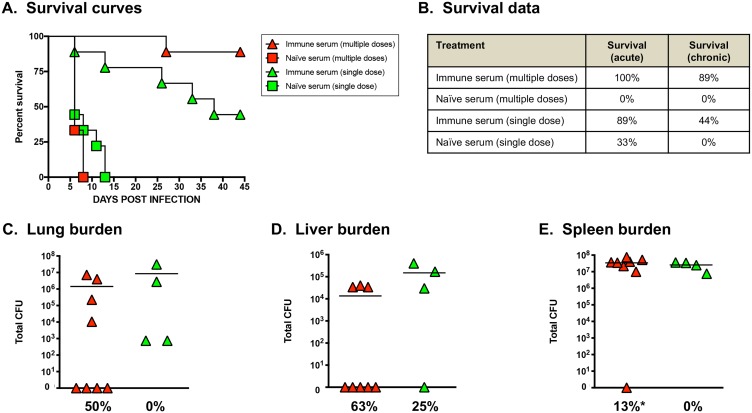
Passive transfer of immune serum provides protective immunity against challenge with a lethal dose of wild-type *B. mallei*. Groups of naive female BALB/c mice were vaccinated with *B. mallei batA* LAS (41) and exsanguinated 30 to 45 days postvaccination, and serum samples from these animals were pooled. Naive female BALB/c mice (7 weeks of age; *n *=* *9 per group) were then injected intraperitoneally with 1 ml of pooled immune sera and challenged 48 h later with 5 LD_50_ of wild-type *B. mallei* ATCC 23344 bacteria via the aerosol route using a Microsprayer device (25); controls consisted of age- and weight-matched naive mice injected with 1 ml of naive serum. Some animals received only a single dose of serum prior to infection, while others also received additional doses on days 7 and 14 postchallenge. Infected animals were monitored daily for signs of illness and morbidity. (A) Kaplan-Meier survival curves. (B) Survival data during the acute (days 1 through 10 postchallenge) and chronic (days 11 through 45 postchallenge) phases of infection. (C to E) Tissues were collected from survivors, homogenized, diluted, and spread on agar plates to determine bacterial loads. Symbols represent the values for individual animals; horizontal bars show mean total CFU for each group. The values below the groups in panels C to E show the percentages of animals that cleared bacteria from tissues from the different groups. The asterisk indicates that one mouse cleared bacteria from all three tissues.

Given their ability to thrive intracellularly, it has been proposed that a vaccine for *B. pseudomallei* and *B. mallei* should primarily generate robust cellular immune responses to eliminate infected host cells and reduce the risk of chronic disease (16, 22, 28, 31–34). However, the data reported by Khakhum et al. (30) indicate that agent-specific CD4^+^ and CD8^+^ T cells play a minor role in protection. These findings are consistent with previous studies demonstrating the importance of antibodies in protection against melioidosis and glanders. For example, vaccination with the *B. pseudomallei purM* LAS Bp82 was shown to provide high levels of protection against lethal intranasal challenge with wild-type *B. pseudomallei* isolate 1026b in BALB/c and C57BL/6 mice (35). Passive transfer of immune serum (elicited by vaccination with Bp82) to BALB/c mice resulted in survival rates of ∼40%, and vaccination of mice lacking B cells with Bp82 did not protect against challenge with wild-type organisms (35). Passive transfer of immune serum elicited by vaccination with *B. pseudomallei* 1026b outer membrane vesicles was shown to provide 80% survival in BALB/c mice against heterologous lethal challenge with wild-type *B. pseudomallei* K96243 (36), and monoclonal antibodies targeting LPS passively protected BALB/c mice against lethal aerosol infection with wild-type *B. mallei* strain ATCC 23344 (37). In addition, hyperimmune sera from horses vaccinated with mallein extract have been successfully used to treat human patients with glanders (38–40). Published work by our group also demonstrated that passive transfer of antibodies elicited by vaccination with *B. mallei* ATCC 23344 *batA* LAS protects against lethal aerosol challenge with homologous wild-type *B. mallei* organisms as well as lethal exposure to multiple wild-type *B. pseudomallei* strains in BALB/c and C57BL/6 mice (41). Importantly, passive transfer of antibodies (elicited by vaccination with *B. mallei batA* LAS) results in dose-dependent, high rates of bacterial clearance from target organs (41) (Fig. 1).

In summary, the report by Khakhum and colleagues (30) complements prior published studies and expands upon them to demonstrate that antibodies are sufficient to protect against lethal aerosol infection with *B. pseudomallei* and *B. mallei*. Future work investigating the kinetics, quality, levels, and functionality of antibody responses in mice vaccinated with highly protective LAS will help drive the melioidosis and glanders vaccine field forward and will provide a powerful platform to identify high-value *Burkholderia* target antigens for the development of countermeasures.
